# Dynamic Model Selection in a Hybrid Ensemble Framework for Robust Photovoltaic Power Forecasting

**DOI:** 10.3390/s25144489

**Published:** 2025-07-19

**Authors:** Nakhun Song, Roberto Chang-Silva, Kyungil Lee, Seonyoung Park

**Affiliations:** Department of Applied Artificial Intelligence, Seoul National University of Science and Technology, 232 Gongneung-ro, Nowon-gu, Seoul 01811, Republic of Korea; nhsong@seoultech.ac.kr (N.S.); rjavierch@seoultech.ac.kr (R.C.-S.); leedake@seoultech.ac.kr (K.L.)

**Keywords:** solar forecasting, hybrid ensemble, meta-learning, meta-modeling, solar energy systems, renewable integration, prediction error analysis

## Abstract

**Highlights:**

**What are the main findings?**

- A flexible hybrid ensemble is proposed for photovoltaic (PV) power forecasting.
- The ensemble dynamically selects among diverse models for each prediction instance.

**What is the implication of the main finding?**

- The model achieves state-of-the-art performance in both accuracy and robustness.
- Evaluation on four real PV plants in South Korea shows strong generalization across different test sizes and CV splits.

**Abstract:**

As global electricity demand increases and concerns over fossil fuel usage intensify, renewable energy sources have gained significant attention. Solar energy stands out due to its low installation costs and suitability for deployment. However, solar power generation remains difficult to predict because of its dependence on weather conditions and decentralized infrastructure. To address this challenge, this study proposes a flexible hybrid ensemble (FHE) framework that dynamically selects the most appropriate base model based on prediction error patterns. Unlike traditional ensemble methods that aggregate all base model outputs, the FHE employs a meta-model to leverage the strengths of individual models while mitigating their weaknesses. The FHE is evaluated using data from four solar power plants and is benchmarked against several state-of-the-art models and conventional hybrid ensemble techniques. Experimental results demonstrate that the FHE framework achieves superior predictive performance, improving the Mean Absolute Percentage Error by 30% compared to the SVR model. Moreover, the FHE model maintains high accuracy across diverse weather conditions and eliminates the need for preliminary validation of base and ensemble models, streamlining the deployment process. These findings highlight the FHE framework’s potential as a robust and scalable solution for forecasting in small-scale distributed solar power systems.

## 1. Introduction

Despite the continuous rise in global electricity demand, the environmental concerns associated with fossil fuel consumption are becoming increasingly critical [1]. This has driven a growing global interest in sustainable energy alternatives, including solar, wind, hydro, and tidal power, collectively termed renewable energy sources (RESs) [2,3]. Among these, solar energy stands out due to its widespread availability and suitability for deployment in urban environments [4]. Recent technological advances have significantly improved the conversion efficiency of photovoltaics (PV), positioning solar energy as one of the most rapidly adopted RES options [5,6].

As a result, solar power generation (SPG) has maintained its status as the fastest-growing electricity source for the 18th consecutive year, with a 24% increase in capacity compared to 2021, reaching approximately 861 GW globally [7]. However, the inherently variable nature of solar energy presents unique challenges for the integration of the power system [8]. SPG output is highly sensitive to meteorological conditions, leading to significant fluctuations in electricity production. Consequently, accurate solar power forecasting is essential to reduce uncertainty, improve economic viability, and ensure reliable power grid operation through efficient integration of renewable sources [9].

Forecasting in SPG is essential for effective grid integration and is commonly categorized into four primary approaches: physical, statistical, machine learning (ML), and hybrid or ensemble methods [10,11]. In addition to methodological classification, forecasts are also distinguished by their temporal horizon—typically divided into short-term (1 h to 1 week), medium-term (1 week to 1 month), and long-term (beyond 1 month) predictions [12]. However, it is important to note that these definitions are not universally standardized. For instance, some studies propose a four-tier classification: very short-term (up to 1 day), short-term (up to 2 weeks), medium-term (up to 3 years), and long-term (up to 30 years) [13,14]. This variation highlights the lack of consensus in the literature regarding forecasting horizon definitions.

Physical models rely on mathematical modeling and Numerical Weather Prediction (NWP) systems to simulate the physical processes affecting solar irradiance. These models incorporate variables such as temperature, atmospheric pressure, solar angles, and cloud cover [15,16]. They are particularly effective for long-term and large-scale forecasting but require high-resolution environmental data and significant computational resources. Representative examples include radiative transfer models, the P-persistent model, and satellite-based forecasting systems [17,18,19,20]. Despite their accuracy, physical models often face challenges related to calibration, spatial resolution, and model complexity [21].

Statistical approaches use historical data to identify patterns and trends in solar power output [22]. Common techniques include autoregressive (AR) and moving average (MA) techniques, and their extensions such as ARIMA and SARIMA, which address non-stationarity through differencing [23,24,25,26]. While these models are computationally efficient and relatively easy to implement, they are fundamentally limited by their reliance on linear assumptions and their inability to adapt to rapidly changing environmental conditions.

In the context of SPG, where input variables such as irradiance, temperature, and cloud cover exhibit strong non-linear and non-stationary behavior, statistical models often fail to capture the underlying dynamics accurately [27,28]. Moreover, traditional statistical models are typically static in nature, i.e., they do not incorporate mechanisms for dynamically adjusting to new data patterns or site-specific characteristics. This rigidity makes them unsuitable for distributed solar power systems, where forecast conditions can vary significantly between locations.

Recent advancements in power systems and the exponential growth of data availability have positioned ML and deep learning (DL) as powerful tools for addressing the non-linearity inherent in the environmental variables affecting SPG forecasting [29,30]. Regression-based, tree-based, and ensemble models have demonstrated strong performance across various locations and forecasting horizons [31,32,33].

DL models, particularly neural networks such as Recurrent Neural Networks (RNNs) and Convolutional Neural Networks (CNNs), have shown promising results in time series forecasting tasks [34,35]. RNNs are well-suited for sequential data, while CNNs are effective in capturing long-term dependencies [36]. However, the performance of these models is highly dependent on the quality and characteristics of the training data [37]. Variability in forecast accuracy often arises when models are applied across datasets from different PV plants, due to differences in module characteristics, data resolution, and environmental conditions [38].

To overcome the limitations of individual models, hybrid or ensembling approaches that integrate physical, statistical, ML, and DL methods have gained prominence in SPG prediction and forecasting [39,40,41]. These methods often employ ensemble techniques such as boosting [42], bagging [43], and stacked generalization (meta-learning) [44] to combine the strengths of diverse models and reduce prediction uncertainty [45,46]. Boosting and bagging improve performance by reweighting or aggregating predictions from multiple models. Meta-learning in particular trains a meta-model on the outputs of several base models, leveraging their complementary strengths while reducing bias and variance [47,48]. Stacking uses cross-validation (CV) to generate meta-features, while blending relies on a hold-out validation set. Though similar in principle, their key distinction lies in how training data are allocated.

Nevertheless, the incorrect model selection can significantly compromise SPG forecasting accuracy due to the unique sensitivities of each algorithm, making it crucial to use a model that best fits the given data. Thus, numerous hybrid models have been proposed in this field. For instance, a day-ahead forecasting model combining wavelet transformation, support vector machines (SVM), and particle swarm optimization (PSO) has shown improved accuracy [49]. Other examples include multi-model ensembles that integrate statistical models with artificial neural networks (ANN) using numerical weather data [50], and advanced ML-only hybrids such as ANN-XGBoost-Ridge regression ensembles or the Transformer-LUBE-GRU framework for deterministic day-ahead forecasting [51,52].

Recent studies have continued to refine deep learning architectures for solar forecasting. Transformer-based models in particular have shown strong performance in capturing long-term dependencies in environmental time series [53,54]. Graph neural networks (GNNs) have also emerged as a promising approach for modeling spatial–temporal relationships across multiple power plants or sensor locations [55]. In parallel, optimization techniques such as evolutionary algorithms and attention-based adaptive ensembling have been explored to further improve hybrid model performance [56].

Despite their advantages, hybrid models are not without limitations. They may inherit the weaknesses of their constituent models, particularly when less accurate models are included in the ensemble [33]. Moreover, hybrid systems often rely on pre-tested base models, reducing flexibility and adaptability [57]. Even when individual models perform well, their combination does not guarantee improved results, necessitating careful evaluation of the ensemble’s overall performance [58].

These limitations are particularly pronounced in the context of SPG forecasting. One of the key advantages of solar power plants is their geographical flexibility, as they can be deployed in a wide range of environments—including urban, rural, and remote areas—without the spatial and scale constraints typically associated with other energy sources [4]. However, this flexibility introduces significant forecasting challenges due to the resulting heterogeneity in environmental conditions and solar exposure.

Each solar power plant operates under distinct meteorological and operational contexts, which leads to variability in the availability, resolution, and quality of the input data. For example, the types and frequency of weather measurements—such as solar irradiance, temperature, humidity, and cloud cover—can differ substantially depending on the location and the instrumentation used [59]. Additionally, the data collection methods and infrastructure (e.g., ground-based sensors vs. satellite data) vary across sites, influencing both the selection and performance of forecasting models.

This distributed and heterogeneous nature of SPG systems demands forecasting models that are both highly adaptable and context-aware. Fixed-output or static models often fail to generalize across different sites as they are typically optimized for specific data distributions and environmental conditions. As a result, a hybrid ensemble model that performs well for one solar power plant may not achieve the same level of accuracy when applied to another, even if the underlying modeling framework remains unchanged [60]. These challenges underscore the need for flexible ensemble frameworks capable of dynamically adapting to local data characteristics and operational constraints rather than relying on a one-size-fits-all approach.

An approach that can be tailored to the specific attributes of each site is essential to overcome the challenge of developing a universally effective forecasting solution. The heterogeneity of environmental conditions, data availability, and system configurations across solar power plants makes it difficult for conventional models to generalize effectively. This underscores the need for a flexible and adaptive forecasting framework capable of dynamically adjusting to site-specific characteristics.

To address these challenges, this study proposes a flexible hybrid ensemble (FHE) framework designed to overcome the limitations of traditional hybrid ensemble methods. It reduces reliance on pre-tested model combinations, increases robustness to data variability across different sites, and enables the inclusion of diverse models without compromising overall performance. Furthermore, it adapts dynamically to local environmental and operational conditions, making it particularly well-suited for distributed solar power systems. Key innovations and contributions include the following:

1. Selective Model Utilization: The FHE framework avoids the common pitfall of performance degradation caused by underperforming models by not mandatorily integrating all base models into the final prediction. Instead, it selectively includes only those models that are expected to contribute positively to forecasting accuracy. This eliminates the need for exhaustive pre-testing of individual models and enhances the framework’s adaptability across different sites.
2. Error-Based Meta-Modeling: A central innovation of the FHE framework is its use of an error-informed meta-model. Unlike traditional meta-models that rely solely on base model outputs, the FHE meta-model incorporates both historical prediction errors and environmental variables to estimate the expected performance of each base model under specific conditions. This allows the system to dynamically assess and select the most suitable models for each forecasting instance.
3. Dynamic Base Model Selection: The framework predicts the expected error of each base model for a given input and selects the subset of models most likely to yield accurate predictions. This instance-specific selection enables the ensemble to adapt its configuration in real time, leveraging even models that may perform poorly on average but excel under certain conditions.

The remainder of this paper is organized as follows: Section 2 describes the research design, including the characteristics of the data and the preprocessing steps undertaken, and also details of the development and implementation of the FHE framework, with particular emphasis on the methodology through which the meta-model dynamically selects the optimal base models. Section 3 presents the experimental results and performance evaluation. Finally, Section 4 concludes the study and discusses future research directions.

## 2. Materials and Methods

This section presents the proposed FHE framework for hourly SPG forecasting. The framework enhances traditional hybrid ensemble meta-modeling by introducing a dynamic, error-aware model selection strategy. Unlike conventional approaches where the meta-model passively aggregates predictions from all base models, the FHE framework actively selects the most suitable base models based on forecast-specific meteorological conditions and contextual factors. It comprises several key components: data imputation, preprocessing, feature engineering, and model performance evaluation under different weather conditions.

### 2.1. Meteorological and Solar Power Data Collection

The meteorological and solar power data used in this study were collected from multiple sources to support the development of a robust forecasting framework. Meteorological observations were obtained (data available at http://www.kma.go.kr/, accessed on 15 July 2025) from the Korea Meteorological Administration (KMA), comprising data from 103 Automated Synoptic Observing System (ASOS) stations and 510 Automatic Weather Stations (AWSs) distributed across South Korea [61]. These datasets include key meteorological variables relevant to solar power forecasting, as listed in Table 1. In parallel, historical SPG data were collected on-site from four distinct PV plants to enable hourly forecasting at each location.

**Figure 1:**
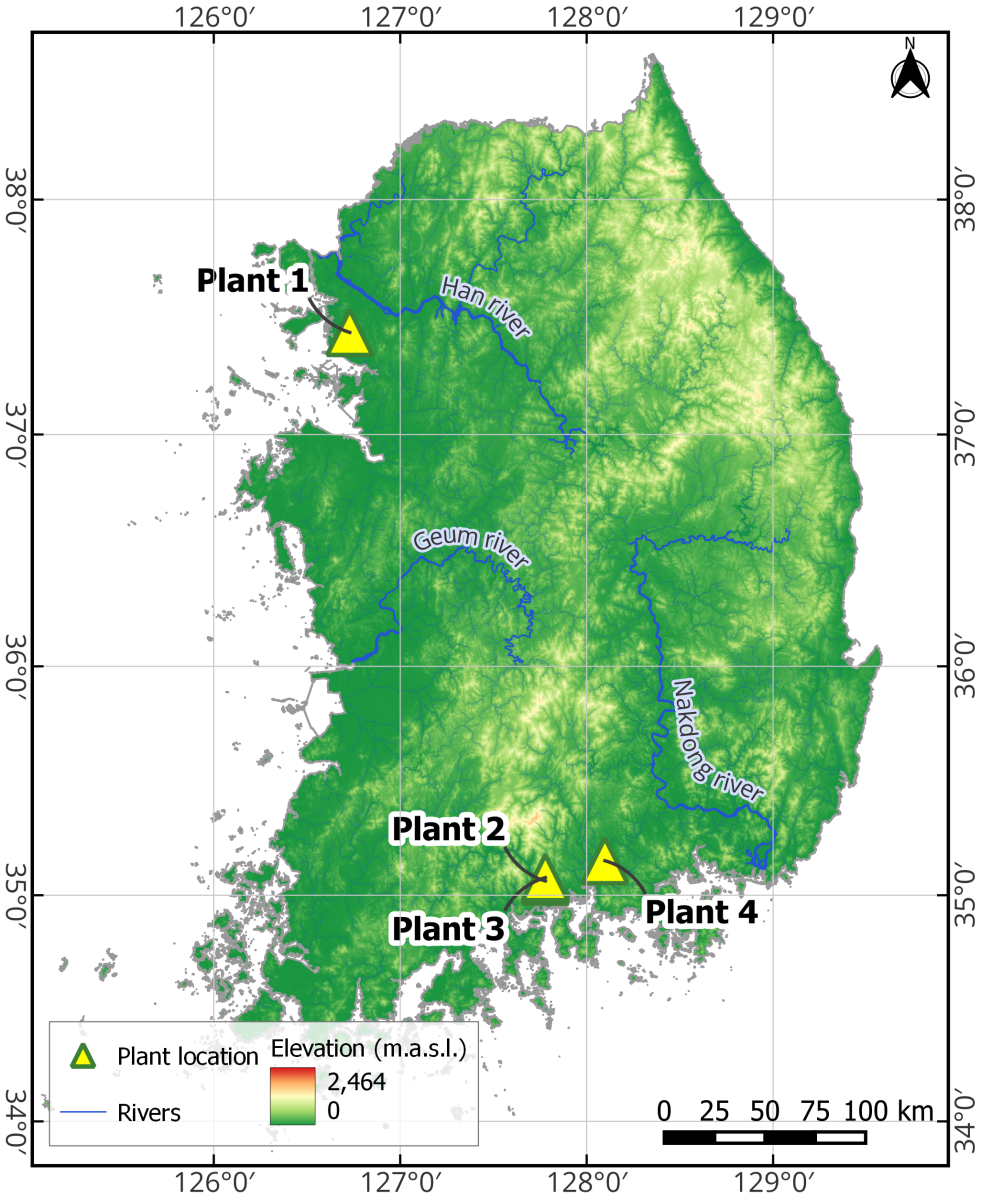
Geographical locations of the four photovoltaic (PV) South Korean plants used in the experimental analysis.

Unlike many previous studies, this work deliberately excludes direct measurements of solar radiation or irradiance from the location site, despite their strong influence on power generation, in order to develop a more universally applicable forecasting model based solely on widely available meteorological data. Specifically, key weather variables were sourced from the ASOS station nearest to each PV plant, with missing values supplemented using data from the more densely distributed AWS network. On average, the distance between each PV plant and its associated ASOS station is approximately 12 km. The geographical locations of the four solar power plants are illustrated in Figure 1. The main characteristics of the four PV plants used in this study are summarized below.

- Plant 1: Data available from January 2019 to June 2022 at 1 h intervals. Capacity: 998 kW.
- Plant 2: Data available from January 2019 to June 2022 at 1 h intervals. Capacity: 369.85 kW.
- Plant 3: Data available from January 2019 to June 2022 at 1 h intervals. Capacity: 48.3 kW.
- Plant 4: Data available from January 2019 to December 2021 at 1 h intervals. Capacity: 905 kW.

### 2.2. Forecasting Hybrid Ensemble: A Modified Framework for Solar Power Prediction

The framework consists of three phases: (1) Data Preparation and Feature Engineering, which includes imputation of missing values, feature scaling, and the creation of derived variables such as temporal encodings and astronomical indicators. (2) Base Model Training and Error Profiling, which involves training a diverse set of base learners—Random Forest (RF), Support Vector Regression (SVR), LightGBM, XGBoost, Transformer, and Multi-Layer Perceptron (MLP) models—and profiling their performance using a customized validation strategy. (3) Meta-Model Learning and Inference, where a meta-model is trained to predict the expected error of each base model for a given input. During inference, the models with the lowest predicted error are selected to generate the final forecast.

**Figure 2:**
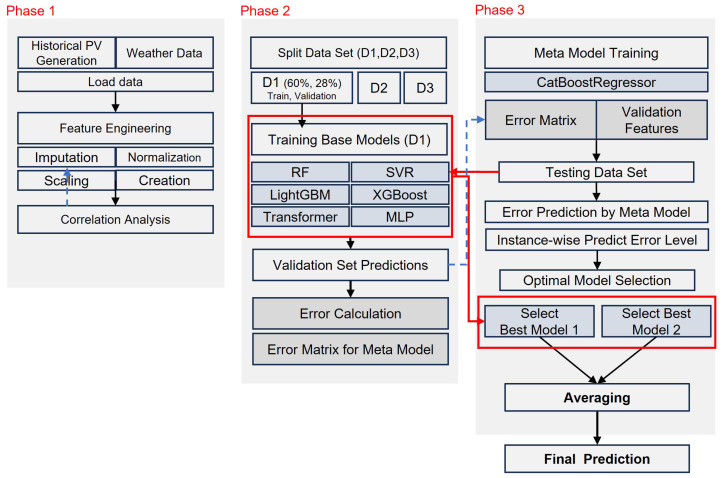
Architecture of the proposed FHE (Forecasting with Heterogeneous Ensembles) framework.

This approach reduces the need for exhaustive pre-selection and allows the inclusion of models that may perform well under specific conditions. A schematic of the FHE workflow is shown in Figure 2.

#### 2.2.1. Data Preparation and Feature Engineering

Prior to model training, the dataset undergoes a series of preprocessing steps aimed at improving data quality and enhancing the predictive power of the input features.

##### Data Scaling

The collected meteorological variables are standardized to ensure consistency across features with varying numerical ranges. This step is essential, as the raw data include variables that span from small decimal values to large magnitudes, which could otherwise introduce bias during model training. Standardization transforms each feature to have a mean of zero and a standard deviation of one, facilitating more stable and efficient learning, particularly for models sensitive to feature scaling.

Given that the base models employed in this study, such as the SVR, Multi-Layer Perceptron (MLP), and Transformer, are sensitive to the scale of input features, this normalization step is critical. The standardization is expressed in Equation (1):

(1) $$D_{i,n}=\frac{D_{i}-\mu \left(D_{\mathrm{train}}\right)}{\sigma (D_{\mathrm{train}})}$$

where $D_{i,n}$ represents the standardized value of the $i^{th}$ data point, $D_{i}$ is the original value, $\mu (D_{\mathrm{train}})$ is the mean of the training dataset, and $\sigma (D_{\mathrm{train}})$ is the corresponding standard deviation.

To ensure comparability across solar power plants of varying capacities, the SPG data are normalized by dividing each power output value by the corresponding rated capacity of the plant. This normalization transforms absolute power values into relative performance metrics, enabling unbiased comparisons across heterogeneous plant configurations. The normalized power generation at time step *i* is defined in Equation (2):

(2) $$P_{i,n}=\frac{P_{i}}{C_{\mathrm{plant}}}$$

where $P_{i,n}$ represents the normalized power output, $P_{i}$ is the actual power generated at time *i*, and $C_{\mathrm{plant}}$ denotes the installed capacity of the respective solar power plant. This scaling approach is essential for accurately evaluating operational efficiency and enhancing the robustness of forecasting model performance across diverse system sizes.

##### Missing Data Imputation

To address missing values in the SPG and meteorological datasets, a hybrid imputation strategy is employed based on the duration of the missing intervals.

- SPG Data Imputation: For gaps shorter than three consecutive hours, linear interpolation is applied due to its simplicity and effectiveness in short-term continuity. For longer gaps, a historical similarity-based imputation method is used. This approach identifies past time points with similar environmental conditions and substitutes the missing SPG values accordingly. The similarity between the missing time step $t_{\mathrm{missing}}$ and a historical candidate $t_{\mathrm{historical}}$ is quantified using a weighted distance metric following Equation (3):
  (3) $$D\left(t_{\mathrm{missing}},t_{\mathrm{historical}}\right)=\sum_{j=1}^{n}w_{j}\;\cdot \;\left|X_{j}\left(t_{\mathrm{missing}}\right)-X_{j}\left(t_{\mathrm{historical}}\right)\right|$$
  where $X_{j}$ denotes the *j*-th environmental variable (e.g., solar radiation, sunshine duration, cosine of the hour angle, elevation angle), and $w_{j}$ is its weight based on correlation with the SPG. The historical time point minimizing this distance follows Equation (4):
  (4) $$t_{\mathrm{best}}=\underset{t_{\mathrm{historical}}}{\mathrm{argmin}}\;D\left(t_{\mathrm{missing}},t_{\mathrm{historical}}\right)$$
  The corresponding SPG value at $t_{\mathrm{best}}$ is then used to impute the missing value. This method leverages domain-specific correlations to enhance imputation accuracy, aligning with practices validated in prior PV forecasting studies [62].
- Filling Gaps in Meteorological Data: To impute missing values in the ASOS meteorological data associated with each PV plant, the Inverse Distance Weighting (IDW) method is employed. This spatial interpolation technique estimates unknown values based on observations from nearby automatic weather monitoring stations located within a 10 km radius of each plant. By assigning greater influence to closer stations, IDW ensures that the interpolated values reflect local weather conditions with higher fidelity. Specifically, a missing value $y(x_{0})$ at location $x_{0}$ is estimated as a weighted average of known values $y(x_{i})$ from surrounding locations $x_{i}$ using Equations (5) and (6):
  (5) $$y\left(x_{0}\right)=\sum_{i=1}^{n}w\left(x_{0},x_{i}\right)\;\cdot \;y\left(x_{i}\right)$$
  (6) $$w\left(x_{0},x_{i}\right)=\frac{d{\left(x_{0},x_{i}\right)}^{-1.7}}{\sum_{i=1}^{n}d{\left(x_{0},x_{i}\right)}^{-1.7}}$$
  where $d(x_{0},x_{i})$ is the Euclidean distance between the target location $x_{0}$ and the known point $x_{i}$. The exponent 1.7 is selected to balance the influence of proximity and spatial variability. This method, widely used in geospatial and meteorological applications, effectively captures local variability and has been validated in numerous studies [63].

Both the SPG and meteorological datasets exhibit a low rate of missing values, with less than 0.2% of the data affected. According to prior studies [64,65,66,67], missing data rates below 5% are generally considered to have a negligible impact on statistical analyses. Nonetheless, to ensure data continuity and maintain the integrity of subsequent modeling efforts, imputation procedures were applied. Given that the primary objective of this study is not to explore advanced preprocessing techniques, straightforward statistical methods were adopted to address the missing values efficiently and transparently [66,67].

##### Feature Space Expansion via Temporal and Astronomical Transformations

To enhance the predictive accuracy of photovoltaic power generation models, this study expanded the feature space by incorporating both astronomical and temporal transformations. These features are designed to capture the physical dynamics of solar movement and the periodic nature of solar irradiance, complementing the meteorological inputs.

The Skyfield Python 1.53 library was used to compute solar azimuth and elevation angles for each PV plant location [68]. Additionally, trigonometric transformations of time variables were applied to encode cyclical temporal patterns.

- Azimuth Angle: The azimuth angle, representing the sun’s horizontal position relative to true north, is calculated by mapping the sun’s celestial coordinates to the local horizon of each plant. This computation accounts for the rotation of the Earth and the apparent daily trajectory of the sun. Atmospheric refraction is also considered to improve the accuracy [69]. The importance of the azimuth angle in PV energy yield has been well established in prior studies, showing its significant influence on annual energy production [70].
- Elevation Angle: The elevation angle, indicating the sun’s vertical position above the horizon, is derived based on the plant’s geographic coordinates and the observation time. Corrections for Earth curvature and atmospheric conditions are included to ensure precision. These angles are critical for modeling solar irradiance on tilted surfaces and are widely used in solar energy modeling [71].
- Temporal Encoding: To capture the inherent periodicity in solar irradiance, time-related features such as the hour of day and month of year are transformed using sine and cosine functions. This approach avoids discontinuities in cyclical variables and enabled the model to learn smooth temporal patterns. The transformation is defined in Equations (7) and (8):
  (7) $${\mathrm{Time}}_{\sin,i}=\sin\left(2\pi \frac{i}{T_{i}}\right)$$
  (8) $${\mathrm{Time}}_{\cos,i}=\cos\left(2\pi \frac{i}{T_{i}}\right)$$
  where *i* denotes the time component (e.g., hour, month), and $T_{i}$ is the total number of units in the cycle (i.e., 24 for hours, 12 for months). This method of encoding cyclical time features has been shown to improve model performance in time series forecasting tasks.

Incorporating these deterministic features derived from astronomical and temporal principles offers a significant advantage over relying solely on meteorological forecasts, which are inherently uncertain [72]. By leveraging physically grounded inputs, the model achieves greater robustness and reliability in forecasting solar power output. A comprehensive list of all the features used in this study, including both meteorological and engineered variables, is provided in Table 1.

#### 2.2.2. Base Model Training and Error Profiling

This subsection outlines the training procedure for the base models and the construction of the meta-model within the FHE framework. A customized data-splitting strategy was employed to prevent data leakage and to ensure that the meta-model was trained on representative error patterns from the base models.

##### Data Splitting

To prevent temporal leakage, the dataset *D* was split chronologically into two primary subsets:

- $D_{1}$ (60%): Used exclusively for training the base models.
- $D_{2}$ (40%): Reserved for error profiling and final evaluation.

While this approach does not explicitly balance the occurrence of seasonal or extreme weather events across splits, it ensures the integrity of the forecasting task and reflects real-world conditions where future events must be predicted from past data.

Subset $D_{2}$ was further divided as follows:

- $D_{3}$ (70% of $D_{2}$): Used to evaluate the performance of each base model and collect errors for training the meta-model.
- Final Test Set (30% of $D_{2}$): Used for the final assessment of the FHE framework.

**Figure 3:**
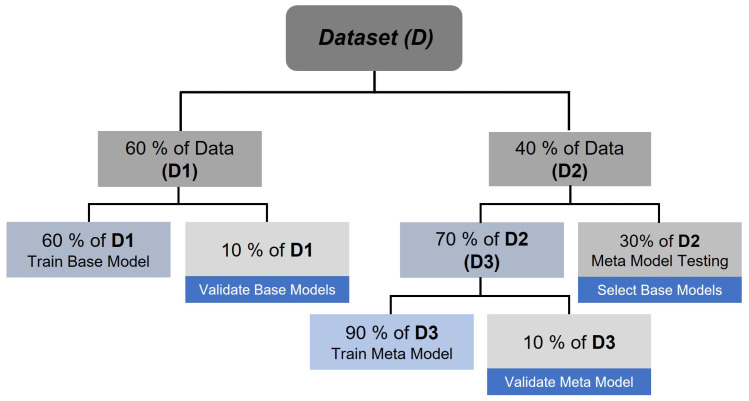
Data partitioning strategy employed in the FHE framework, illustrating training, validation, and test splits.

This structure (Figure 3) ensures that the meta-model is trained on unseen data relative to the base models, allowing it to learn error patterns without overfitting. The resulting meta-model is then used to select or weight base models dynamically during inference.

##### Base Model Training

The FHE framework integrates a diverse set of ML and DL models commonly used in renewable energy forecasting, including RF, SVR, LightGBM, XGBoost, Transformer, and MLP models [37,73,74,75,76]. Although RNN-based models such as LSTM and GRU have been widely used in time series forecasting, they were not included in this study for two main reasons: (1) Transformer-based models have shown superior performance in modeling long-range dependencies [77], and (2) RNNs typically require more complex training while offering limited gains compared to both traditional models (e.g., RF, SVR) and modern architectures (e.g., Transformers). All base models are trained using the training subset $D_{1}$. The Transformer model is implemented as a regressor using only the encoder architecture. Input features are embedded into a high-dimensional space, and the encoded representation is passed through a fully connected layer to produce the final output. This design is optimized for tabular regression tasks, where sequence generation is not required. Both the Transformer and MLP models are implemented in PyTorch 2. Details of the model-specific hyperparameters, loss functions, and optimizers are provided in Table 2.

Each base model $M_{i}$ is trained on the training subset $D_{1}$ using input features $X_{\mathrm{train}}$ and corresponding target values $y_{\mathrm{train}}$. Once trained, the model generates predictions ${\hat{y}}_{i,\mathrm{val}}$ on the validation set $X_{\mathrm{val}}$. The prediction error for each instance *j* is then computed as the absolute difference between the predicted and actual values. These individual errors are aggregated to form an error matrix *E*, which captures the performance of all base models across the validation set following Equations (9) and  (10):

(9) $$e_{i,j}=\left|y_{j}-{\hat{y}}_{i,j}\right|$$

(10) $$E=\left[e_{1}\left(X_{\mathrm{val}}\right),e_{2}\left(X_{\mathrm{val}}\right),\ldots ,e_{K}\left(X_{\mathrm{val}}\right)\right]$$

Here, *K* denotes the total number of base models. This matrix serves as the foundation for training the meta-model, which learns to identify the most suitable base models for each input instance based on historical error patterns. Each power plant is modeled independently, with a separate FHE ensemble trained using its respective historical data. This design reflects the operational independence of each plant, which is subject to unique weather patterns, site-specific configurations, and forecasting challenges.

#### 2.2.3. Meta-Model Learning and Forecast Inference

The final stage of the FHE framework involves training a meta-model to predict the expected error of each base model and using these predictions to guide model selection during inference. This process is based on the error matrix generated from the validation subset $D_{3}$, along with the corresponding input features.

The meta-model, implemented using the CatBoost regressor, is trained to learn the relationship between input features and the prediction errors of each base model. CatBoost was selected for its ordered boosting mechanism and its ability to handle categorical features effectively through target-based statistics. Hyperparameter optimization was performed using the Optuna library [78]. Once trained, the meta-model is applied to the test set (30% of $D_{2}$) to estimate the expected error ${\hat{E}}_{\mathrm{test}}$ for each base model, and, for each test instance, the two base models with the lowest predicted errors are selected. Their predictions are averaged to produce the final forecast following Equations (11) and (12). The top two base models are selected dynamically for each instance based on their predicted error. This choice offers a trade-off between robustness (by leveraging model diversity) and simplicity (avoiding excessive aggregation that may introduce noise). Although the number of selected models could be treated as a tunable hyperparameter, it was fixed to two in this work to maintain interpretability and focus the analysis.

(11) $${\hat{E}}_{\mathrm{test}}=M_{\mathrm{meta}}\left(X_{\mathrm{test}}\right)$$

(12) $${\hat{y}}_{\mathrm{final},j}=\frac{1}{2}\sum_{i\in S_{j}}{\hat{y}}_{i,j}$$

Here, $S_{j}$ denotes the set of the two base models with the lowest predicted errors for instance *j*, and ${\hat{y}}_{i,j}$ is the prediction from base model *i*. This dynamic selection mechanism allows the FHE framework to adaptively leverage the strengths of different models under varying conditions, improving both accuracy and robustness.

The meta-model is trained to estimate the error that each base model is expected to incur given a particular input configuration. During inference, it still receives only the input features and outputs a predicted error for each base model. The two base models with the lowest predicted errors are selected, and their outputs are averaged to produce the final forecast. This approach enables dynamic model selection based on the learned performance patterns of the base models, even in the absence of true target values. The complete algorithmic flow of the FHE framework is summarized in Algorithm 1.
**Algorithm 1** FHE framework: Forecasting with Heterogeneous Ensembles for photovoltaic power prediction.1:**Input:** Dataset $D\to \{D_{1},D_{2}\}$, with $D_{2}\to \left\{D_{3},D_{\mathrm{test}}\right\}$2:**Output:** Forecasts ${\left\{{\hat{y}}_{\mathrm{final},j}\right\}}_{j=1}^{|D_{\mathrm{test}}|}$3:**Train base models** $M={\left\{M_{i}\right\}}_{i=1}^{K}$ on $D_{1}$4:**for** each $M_{i}\in M$, $\left(x_{j},y_{j}\right)\in D_{3}$ **do**5:   ${\hat{y}}_{i,j}\leftarrow M_{i}\left(x_{j}\right)$,    $e_{i,j}\leftarrow \left|y_{j}-{\hat{y}}_{i,j}\right|$6:**end for**7:Construct $E\in R^{K\;\times \;|D_{3}|}$ with $E_{i,j}=e_{i,j}$8:**Train meta-model** $M_{\mathrm{meta}}:R^{d}\to R^{K}$ on ${\left\{\left(x_{j},E_{:,j}\right)\right\}}_{j=1}^{|D_{3}|}$9:**for** each $x_{j}\in D_{\mathrm{test}}$ **do**10:   ${\hat{E}}_{j}\leftarrow M_{\mathrm{meta}}\left(x_{j}\right)=\left[{\hat{e}}_{1,j},\ldots ,{\hat{e}}_{K,j}\right]$11:   $S_{j}\leftarrow \underset{|S|=2}{\arg\min S\subset \{1,\ldots ,K\}}\sum_{i\in S}{\hat{e}}_{i,j}$12:   ${\hat{y}}_{\mathrm{final},j}\leftarrow \frac{1}{2}\sum_{i\in S_{j}}M_{i}\left(x_{j}\right)$13:**end for**14:**Return:** ${\left\{{\hat{y}}_{\mathrm{final},j}\right\}}_{j=1}^{|D_{\mathrm{test}}|}$

### 2.3. Evaluation Metric

To assess the short-term PV forecasting performance, several statistical metrics were employed: the Mean Squared Error (MSE), the Root Mean Squared Error (RMSE), the coefficient of determination ($R^{2}$), the Normalized Mean Absolute Percentage Error (NMAPE), and the MAE [16,22,36,60]. Additionally, the Normalized MAE (NMAE), as utilized in the Korea Power Exchange (KPX) renewable generation forecasting system, was included to calculate errors for outputs exceeding 10% of the installed capacity. These metrics are defined in Equations (13)–(18):

(13) $$\mathrm{MSE}=\frac{1}{n}\sum_{i=1}^{n}{\left(y_{i}-{\hat{y}}_{i}\right)}^{2}$$

(14) $$\mathrm{RMSE}=\sqrt{\frac{1}{n}\sum_{i=1}^{n}{\left(y_{i}-{\hat{y}}_{i}\right)}^{2}}$$

(15) $$R^{2}=1-\frac{\sum_{i=1}^{n}{\left(y_{i}-{\hat{y}}_{i}\right)}^{2}}{\sum_{i=1}^{n}{\left(y_{i}-\bar{y}\right)}^{2}}$$

(16) $$\mathrm{MAPE}=\frac{1}{n}\sum_{i=1}^{n}\left|\frac{y_{i}-{\hat{y}}_{i}}{y_{i}}\right|\times 100$$

(17) $$\mathrm{MAE}=\frac{1}{n}\sum_{i=1}^{n}\left|y_{i}-{\hat{y}}_{i}\right|$$

(18) $$\mathrm{NMAE}(\%)=\frac{1}{n}\sum_{\begin{array}{c} i=1\\ y_{i}\geq 0.1 \end{array}}^{n}\left|\frac{y_{i}-{\hat{y}}_{i}}{y_{i}}\right|\times 100$$

## 3. Results and Discussion

This section evaluates the performance of the proposed FHE framework across four PV plants with capacities ranging from 48.3 kW to 998 kW. The results are reported as the average of 10 independent runs (epochs). Performance comparisons are made against standard models and ensemble baselines using multiple error metrics.

### 3.1. Effect of Feature Engineering on Base Model Accuracy

The impact of engineered features on forecasting accuracy was assessed by comparing four feature scenarios across six base models (RF, SVR, LGBM, XGB, Transformer, and MLP) and four PV plants with varying capacities. The results, averaged over 10 runs, are summarized in Table 3 using the error metrics MSE, NMAE, and $R^{2}$. The assessment included four input configurations, each combining the meteorological data with a different subset of features:

1. **Scenario 1:** Meteorological + Radiation + Engineered Features;
2. **Scenario 2:** Meteorological + Engineered Features (No Irradiance);
3. **Scenario 3:** Meteorological (No Irradiance, No Engineered Features);
4. **Scenario 4:** Meteorological + Irradiance (No Engineered Features).

Across all plants and models, the combination with meteorological data, irradiance, and engineered features (Scenario 1) consistently delivered the best performance. This suggests that the inclusion of solar position (azimuth, elevation) and temporal encodings (hour, month) effectively enhances the model’s ability to capture diurnal and seasonal variability. For instance, the Random Forest achieved an $R^{2}$ above 0.86 at all plants, with particularly strong performance at Plants 2 and 4 ($R^{2}$ = 0.897 and 0.898, respectively). Among all models, the LGBM slightly outperformed the others on average, showing a lower MSE and NMAE at most plants.

When direct irradiance measurements were unavailable (Scenario 2), engineered features still contributed significantly to preserving model accuracy. For instance, RF performance was only slightly degraded compared to in Scenario 1, with the $R^{2}$ dropping by 1–3 percentage points at most plants. This suggests that the engineered features can partially compensate for the absence of irradiance by implicitly capturing solar geometry and temporal dynamics. Interestingly, at Plant 4, the MLP outperformed all other models with an $R^{2}$ = 0.860, highlighting its robustness when dealing with incomplete input modalities.

Scenario 3 presented a clear degradation in performance, confirming the critical role of both irradiance and engineered features. All models experienced substantial increases in MSE and NMAE, with $R^{2}$ values dropping dramatically. For example, the $R^{2}$ for RF at Plant 1 fell from 0.869 (Scenario 1) to 0.255. This trend was consistent across all models and plants, underscoring that meteorological data alone are insufficient for accurate power prediction, especially when solar geometry is not explicitly encoded.

In Scenario 4, the removal of engineered features led to moderate performance loss compared to Scenario 1, yet the models still performed better than in Scenario 2 in most cases due to the presence of irradiance data. The RF model showed a noticeable drop in $R^{2}$ at Plant 1 (from 0.869 to 0.833), but maintained reasonable accuracy overall. These results indicate that, while irradiance is a strong predictor, engineered features provide an additional signal that boosts model generalization, especially in edge cases (e.g., early mornings or cloudy days).

To assess the role of irradiance and engineered features, we compared four feature configurations across all models and plants. Comparing Scenario 1 (all features) to Scenario 2 (engineered features only) revealed only a slight drop in performance, indicating that engineered features can effectively compensate for missing irradiance. For example, the MLP model at Plant 1 maintained nearly identical $R^{2}$ values (0.853 vs. 0.852), suggesting these features successfully capture relevant solar geometry and temporal patterns. A more dramatic change appeared between Scenario 2 and Scenario 3 (neither irradiance nor engineered features), where performance degraded substantially. The RF model at Plant 1, for instance, dropped from 0.848 to 0.255, confirming that engineered features are critical when irradiance is unavailable.

Comparing Scenario 3 to Scenario 4 (irradiance only), we found that irradiance improved performance but not as much as engineered features. For instance, SVR at Plant 2 improved from 0.198 to 0.852, yet similar or better performance is often achieved with engineered features alone. The comparison between Scenario 1 and Scenario 4 shows that, even when irradiance is available, engineered features still offer additional predictive value. Ensemble models like LGBM and XGB benefited the most, with consistent performance gains across plants.

Finally, engineered features are essential for maintaining accuracy in the absence of irradiance and remain beneficial even when irradiance is present, underscoring their value in robust solar energy forecasting.

### 3.2. Comprehensive Performance Analysis of the FHE Framework

The proposed FHE framework was evaluated using real operational data from PV plants to assess its forecasting accuracy. The analysis focused on the framework’s ability to dynamically select and integrate forecasts from multiple base models based on real-time weather conditions, thereby enhancing prediction reliability. Three baseline settings were considered:

1. Baseline 1: Each base model was evaluated independently within the FHE framework, providing a reference for standalone performance.
2. Baseline 2: Conventional hybrid ensemble strategies were applied, including meta-modeling and bagging, which combine forecasts from all base models.
3. Baseline 3 (FHE): The proposed FHE approach selectively integrates forecasts from the best-performing model for specific conditions, optimizing prediction dynamically.

**Figure 4:**
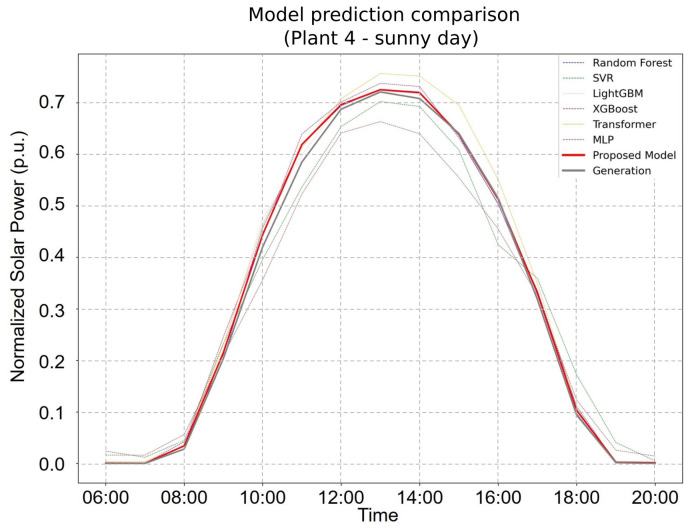
Forecasting performance of the proposed FHE model on a sunny day at Plant 4. The model accurately captures peak generation periods and overall power output trends.

**Figure 5:**
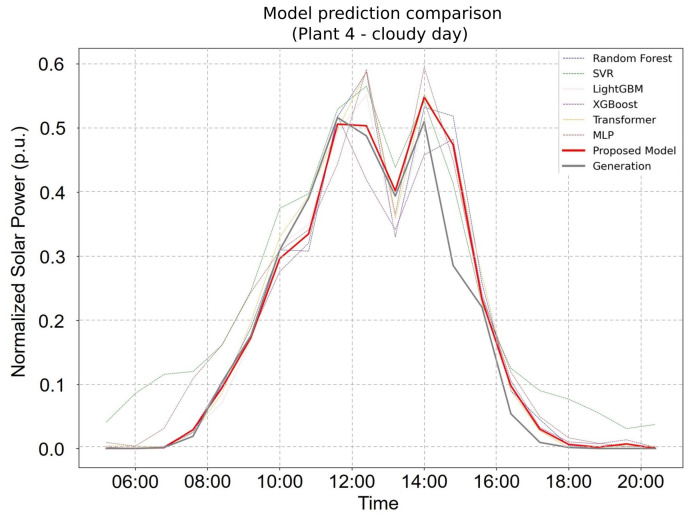
Forecasting performance of the proposed FHE model on a non-sunny (cloudy or overcast) day at Plant 4. The model effectively tracks power generation variability under less predictable conditions.

Figure 4 and Figure 5 further illustrate the FHE framework’s forecasting capability. On clear days (Figure 4), the proposed model not only achieved the highest overall accuracy but also captured peak solar production values with superior precision. Under cloudy conditions (Figure 4), it continued to perform reliably, closely tracking the fluctuations in actual power generation. These findings underscore the FHE framework’s adaptability and robustness across diverse environmental conditions, with it consistently outperforming individual models and conventional ensembles in both stable and volatile weather scenarios.

Table 4 presents a comprehensive evaluation of the proposed FHE model against individual base models and traditional ensemble approaches (bagging and meta-modeling) across four PV plants with varying capacities and operational contexts.

Across all plants and performance metrics, the proposed FHE consistently outperformed all baseline and ensemble methods. Notably, for Plant 1, the FHE achieved the lowest RMSE (65.785 kW), MAE (43.470%), and MAPE (12.923%) while also yielding the highest $R^{2}$ (0.864), indicating superior fit and error minimization. A similar pattern was observed for Plant 2, where the FHE attained an $R^{2}$ of 0.906 and an MAPE of just 12.332%, outperforming even the most competitive baselines such as the RF and LGBM. The performance gains were especially remarkable at Plant 4, a large-scale system with complex dynamics. The FHE achieved a significant reduction in RMSE (55.568 kW vs. >59 kW for all others), along with a notably lower MAE (36.535%) and the highest $R^{2}$ (0.902), highlighting its robustness in high-capacity scenarios. At Plant 3, although the magnitude of errors was smaller due to the lower capacity of the plant, the FHE still achieved the best overall scores, indicating its scalability across plant sizes.

Interestingly, while tree-based models such as RF and LGBM performed competitively, especially at Plants 1 and 2, and bagging provided modest gains, neither traditional ensemble approach matched the adaptive advantage of the FHE. Transformer and MLP models generally lag in performance, particularly in overcast or highly variable conditions, as is evident from their higher RMSE and MAPE values. These results affirm that the selective and condition-aware nature of the FHE model enables it to leverage the strengths of different base learners under varying conditions, thereby achieving more accurate and reliable short-term solar power forecasts across heterogeneous PV plants.

### 3.3. Evaluation of the Robustness of the FHE Framework

To rigorously evaluate the robustness and generalizability of the proposed FHE framework, two complementary experiments were designed: (1) variation of the test set ratios, and (2) time series CV analysis. Initially, a test set ratio of 12% was adopted to capture sufficient temporal variability while maximizing the training data available for the meta-model. To assess the sensitivity of the FHE’s performance to this choice, additional experiments were conducted with increased test set ratios of 16% and 20%. The results of this analysis are summarized in Table 5.

Across all plants, there was a general trend of increasing error as the test set ratio increased. This was expected, as increasing the size of the test set reduces the number of data available for training, which can impair model generalization. However, the extent of performance degradation varied by model, suggesting differences in robustness. The proposed FHE framework consistently achieved the lowest MAPE and NMAE scores across all plants and test set configurations, highlighting its superior generalization and robustness. For instance, at Plant 1, the MAPE increased only modestly from 12.923% (12%) to 15.079% (20%), which is a smaller increment than observed in other models such as the SVR (from 17.328% to 19.786%) or Transformer (from 18.934% to 21.158%).

We considered the results individually for each plant. At Plant 1 (998 kW), the FHE achieved the best MAPE and NMAE scores at all test set sizes, with a strong lead over both individual models and ensemble baselines. Notably, even compared to strong competitors like the LGBM and RF, the FHE provided a relative error reduction of 12–18% in MAPE. At Plant 2 (369.85 kW), again, the FHE outperformed all other models. Interestingly, the gap between the FHE and the others was slightly larger at this plant, with MAPE improvements of up to 20% compared to the SVR or XGB at the 20% test ratio.

In the situation with a low-capacity plant, Plant 3 (48.3 kW) exhibited generally higher errors across all models, likely due to the higher variability in small-scale energy production. However, the FHE remained the top performer, maintaining MAPE scores 1.5–3 points lower than those of other models, which is particularly notable given the plant’s challenging characteristics. Finally, at Plant 4 (905 kW), the FHE once again dominated in terms of performance. Its MAPE remained under 12.8% even at the highest test ratio, while all other models reported MAPE values above 14%, indicating better resilience to reduced training data.

Traditional ensemble methods like bagging and meta-ensembles provide improved robustness over single learners (e.g., RF, SVR), but they are consistently outperformed by the FHE framework. This suggests that the design of the FHE offers a more flexible and effective mechanism for combining base learners under data variability, presumably leveraging adaptive weighting or dynamic ensemble construction. For example, at Plant 4 at the 20% test ratio, the Bagging Ensemble had a 14.255% MAPE, the Meta-Ensemble a 14.978% MAPE, and the FHE a 12.771% MAPE. This performance gap underlines the FHE’s ability to better adapt to structural variations and uncertainties in the input data.

Furthermore, to assess the FHE’s robustness to sequence-dependent biases and its ability to generalize across different temporal segments, a time series split-based CV was performed. Unlike random splits, this approach preserves the temporal order of observations, thereby providing a more realistic simulation of forecasting in unseen future conditions [79]. In this setup, four CV groups were executed, each using a chronologically expanding training window and a test set comprising the most recent 12% of the data. The iterations were defined as follows and the results are presented in Table 6:

- **CV 1:** Training from 1 January 2019 to 13 September 2019;
- **CV 2:** Training from 1 January 2019 to 25 May 2020;
- **CV 3:** Training from 1 January 2019 to 5 February 2021;
- **CV 4:** Training from 1 January 2019 to 18 October 2021.

In CV 1, the FHE outperformed all the models across all four plants. It achieved the lowest MAPE and NMAE, and the highest $R^{2}$ scores, with particularly strong results for Plant 4 (MAPE: 16.565%, $R^{2}$: 0.869), indicating a highly accurate and robust forecast even with a limited training history. Notably, classical machine learning models like the LGBM and RF performed competitively, but consistently lagged behind the FHE in both magnitude of error and explained variance. As the training set increased in CV 2, all models improved in performance, benefiting from more diverse and comprehensive temporal data. However, the margin by which the FHE outperformed others widened. It achieved substantial improvements in accuracy metrics, with Plant 1 showing a remarkable reduction in MAPE to 15.059% and an increase in $R^{2}$ to 0.874. The Bagging Ensemble and Meta-Ensemble provided competitive baselines, demonstrating the effectiveness of aggregation techniques, yet the FHE still maintained an edge, particularly in reducing the systematic under/over-estimation captured by the NMAE.

In CV 3, the gap between the models narrowed somewhat, as more historical variation was captured by the models. Despite this, the FHE continued to lead in all metrics across the board. For instance, at Plant 3, the FHE obtained the best combination of MAPE: 19.743%, NMAE: 6.044%, and $R^{2}$: 0.827, reflecting its resilience in small-capacity scenarios where noise and data imbalance are more pronounced. The Transformer and MLP models showed more volatile performance, suggesting that deep architectures without tailored inductive biases or ensemble mechanisms may overfit or underperform in limited-training contexts.

By the final iteration, CV 4, where the models had the most extensive training data, performances converged, but the FHE retained its leadership. For instance, at Plant 4, the FHE reported an MAPE of 12.976% and an $R^{2}$ of 0.919, exceeding even ensemble models. Interestingly, the RF and LGBM remained consistently strong contenders across all iterations, especially in the early phases, highlighting their robustness and data efficiency. However, their relative gains diminished as the data complexity increased. The SVR and MLP consistently underperformed, particularly in larger-capacity plants, likely due to their limited ability to capture non-linearities or contextual dependencies across temporal segments.

## 4. Conclusions

This study conducted a comprehensive comparative analysis of the proposed FHE model against a wide range of baseline methods for short-term PV power forecasting. The evaluation spanned four real-world PV plants of varying capacities and employed a robust shuffle-based cross-validation strategy across different temporal windows. The results demonstrate that the FHE model consistently outperformed all baseline models, including traditional regressors (RF, SVR, LightGBM, XGBoost), DL architectures (MLP, Transformer), and ensemble methods (Bagging Ensemble and Meta-Ensemble), across all evaluation metrics.

Notably, the FHE model exhibited a superior forecasting accuracy, reflected by the lowest MAPE and NMAE values across all plants and cross-validation splits; a high explanatory power, achieving the highest $R^{2}$ scores consistently, indicating a strong ability to capture the underlying data variance; and robust generalization, with stable performance across different time splits and plant sizes, including challenging cases such as small-scale PV installations with higher noise and variability. While ensemble methods such as the Bagging Ensemble and Meta-Ensemble improved upon individual traditional models, they were still outperformed by the FHE, underscoring the effectiveness of its hybrid design. Furthermore, DL models underperformed relative to both ensemble and tree-based methods, suggesting that their complexity may not be fully leveraged in this context, potentially due to data limitations or architecture mismatch.

Another important observation is that forecasting performance tends to degrade for smaller plants, likely due to increased stochasticity in their power generation profiles. Nevertheless, the FHE model maintained its lead even under these more difficult conditions, further reinforcing its versatility and robustness. In conclusion, the FHE model emerges as a reliable, accurate, and scalable solution for PV power forecasting across diverse operating conditions. These promising results suggest its strong potential for deployment in operational settings, where accurate and generalizable energy forecasts are critical for grid stability and energy management.

Interesting directions for future research are the application of transfer learning between plants with similar characteristics and the use of federated learning frameworks to allow collaborative model training without centralized data aggregation. Also, we aim to explore other sequential-based architectures, such as Recurrent Neural Networks (RNNs), which are specifically designed for long-term time series forecasting. Additionally, another research path is to investigate the impact of selecting different numbers of base models in the final ensemble. While this study fixed the number to two for simplicity and interpretability, alternative strategies such as adaptive top-*k* selection or error-weighted combinations may offer additional improvements in robustness and accuracy.

## Figures and Tables

**Table 1:** Summary of input features used for solar power generation (SPG) forecasting, categorized by source and feature type.

Features (Units)	ASOS	AWS	Engineered Features	Plant Data
Solar Power Generation (kW)				✓
Temperature (°C)	✓	✓		
Rainfall (mm)	✓	✓		
Wind Speed (m/s)	✓	✓		
Wind Direction (16 cat.)	✓			
Humidity (%)	✓	✓		
Steam Pressure (hPa)	✓	✓		
Site Pressure (hPa)	✓	✓		
Dew Point (°C)	✓			
Sunshine Duration (h)	✓			
*Solar Irradiance (MJ/m^2^)*	✓			
Visibility (×10 m)	✓			
*Azimuth (°)*			✓	
*Elevation (°)*			✓	
*Time Sin*			✓	
*Time Cos*			✓	

**Table 2:** Hyperparameters and initialization strategies used for each model in the experimental setup.

Parameter	Value/Method
MLP Hidden Layers	10
Transformer Layers	3
Transformer Heads	4
Batch Size	32
Learning Rate	$3\times 10^{-3}$
Loss Function	MAE
Optimizer	AdamW
Learning Rate Scheduler	ReduceLROnPlateau
Initialization Method	He
Early Stopping	10 epochs

**Table 3:** Performance metrics of individual base models across different scenarios and four PV plants.

	Plant 1 (Capacity: 998 kW)				Plant 2 (Capacity: 369.85 kW)				Plant 3 (Capacity: 48.3 kW)				Plant 4 (Capacity: 905 kW)		
**Model**	**MSE (kW, ↓**)	**NMAE (%, ↓)**	$R^{2}$ (↑)		**MSE (kW, ↓)**	**NMAE (%, ↓)**	$R^{2}$		**MSE (kW, ↓)**	**NMAE (%, ↓)**	$R^{2}$ (↑)		**MSE (kW, ↓)**	**NMAE (%, ↓)**	$R^{2}$ (↑)
Scenario 1: Meteorological Data + Radiation + Engineered Features															
RF	4.016	4.161	0.869		1.786	**4.325**	0.897		**0.362**	5.124	**0.859**		**3.341**	**3.784**	**0.898**
SVR	4.364	4.690	0.858		2.006	5.413	0.885		0.364	6.004	0.858		3.930	4.888	0.880
LGBM	**3.998**	**4.198**	**0.870**		**1.788**	4.493	**0.897**		0.365	**5.305**	0.857		3.427	3.790	0.896
XGB	4.825	4.647	0.843		1.963	4.775	0.887		0.401	5.750	0.843		3.961	4.046	0.879
Transformer	6.456	5.312	0.789		2.192	4.828	0.874		0.411	5.756	0.840		3.843	4.270	0.883
MLP	4.544	4.915	0.852		1.925	5.272	0.889		0.394	5.867	0.846		3.224	3.882	0.902
Scenario 2: Meteorological Data + Engineered Features (No Irradiance)															
RF	4.682	4.630	0.848		**2.117**	**4.912**	0.888		0.473	7.001	0.815		**4.463**	**4.558**	**0.864**
SVR	5.128	5.200	0.823		2.692	6.094	0.867		0.479	7.498	**0.813**		4.574	5.344	0.861
LGBM	4.627	4.613	0.849		2.279	4.966	0.889		**0.458**	7.035	0.821		4.501	4.596	0.863
XGB	6.114	5.308	0.800		2.472	5.265	0.877		0.460	**6.988**	0.820		4.881	4.950	0.851
Transformer	8.001	6.087	0.739		3.196	6.000	0.828		0.619	8.434	0.758		5.076	5.138	0.845
MLP	**4.504**	**4.835**	**0.853**		2.628	6.051	0.866		0.578	8.542	0.774		4.607	5.212	0.860
Scenario 3: Meteorological Data (No Irradiance, No Engineered Features)															
RF	22.818	12.028	0.255		11.869	14.316	0.318		2.233	17.715	0.128		19.326	11.708	0.411
SVR	**21.305**	**11.366**	**0.305**		**11.357**	**13.700**	**0.347**		**1.952**	**16.142**	**0.238**		**18.365**	**11.296**	**0.440**
LGBM	22.271	11.852	0.273		11.568	14.139	0.335		2.201	17.633	0.140		19.095	11.643	0.418
XGB	23.544	12.379	0.232		12.043	14.292	0.308		2.348	18.139	0.083		20.727	11.986	0.368
Transformer	28.878	13.332	0.057		14.643	15.379	0.158		2.574	18.873	0.056		22.502	12.303	0.314
MLP	23.357	12.551	0.238		11.685	14.398	0.337		2.357	18.490	0.079		20.508	12.492	0.375
Scenario 4: Meteorological Data + Irradiance (No Engineered Features)															
RF	5.109	5.080	0.833		1.893	5.080	0.833		0.414	5.860	0.838		**4.009**	**4.618**	**0.878**
SVR	**4.533**	**4.828**	**0.852**		**1.680**	**4.828**	**0.852**		**0.389**	5.988	**0.848**		4.120	4.969	0.874
LGBM	4.931	4.973	0.839		1.828	4.973	0.839		0.409	**5.840**	0.840		4.130	4.673	0.874
XGB	5.883	5.527	0.808		2.180	5.527	0.808		0.437	6.033	0.829		4.856	5.076	0.852
Transformer	7.699	6.466	0.749		2.853	6.466	0.749		0.435	6.097	0.830		5.068	4.999	0.46
MLP	4.965	4.941	0.838		1.840	4.941	0.838		0.433	6.278	0.831		4.037	4.760	0.877

↑ Greater is better. ↓ Lower is better.

**Table 4:** Comparative performance of the proposed FHE model against baseline methods across all PV plants.

	Plant 1 (Capacity: 998 kW)						Plant 2 (Capacity: 369.85 kW)				
**Model**	**RMSE (kW, ↓)**	**MAE (%, ↓)**	$R^{2}$ (↑)	**MAPE (%, ↓)**	**NMAE (%, ↓)**		**RMSE (kW, ↓)**	**MAE (%, ↓)**	$R^{2}$ (↑)	**MAPE (%, ↓)**	**NMAE (%, ↓)**
RF	68.112	46.207	0.848	14.480	4.630		26.865	18.165	0.888	13.721	4.912
SVR	71.535	51.891	0.823	17.328	5.200		29.235	22.538	0.867	16.854	6.094
LGBM	67.957	46.036	0.849	14.773	4.613		26.698	18.367	0.889	14.284	4.966
XGB	78.115	52.975	0.800	16.417	5.308		28.141	19.473	0.877	14.668	5.265
Transformer	89.360	60.747	0.739	18.934	6.087		33.292	22.190	0.828	17.381	6.000
MLP	67.047	48.253	0.853	15.206	4.835		29.379	22.381	0.866	16.534	6.051
Bagging Ensemble	70.896	48.259	0.818	16.969	4.935		27.159	18.222	0.884	14.837	5.227
Meta-Ensemble	75.387	50.242	0.814	15.213	5.034		28.541	19.993	0.873	14.731	5.406
Proposed FHE	**65.785**	**43.470**	**0.864**	**12.923**	**4.356**		**24.837**	**16.700**	**0.906**	**12.332**	**4.686**
	Plant 3 (Capacity: 48.3 kW)						Plant 4 (Capacity: 905 kW)				
RF	4.777	3.381	0.815	18.508	7.001		63.483	41.200	0.864	14.116	4.558
SVR	4.811	3.621	0.813	19.875	7.498		64.265	48.309	0.861	17.645	5.344
LGBM	4.702	3.398	0.821	18.751	7.035		63.754	41.549	0.863	14.447	4.596
XGB	4.714	3.375	0.820	18.677	6.988		66.391	44.745	0.851	15.346	4.950
Transformer	5.469	4.074	0.758	22.701	8.434		67.703	46.501	0.845	16.397	5.138
MLP	5.283	4.126	0.774	21.674	8.542		64.501	47.165	0.860	16.537	5.212
Bagging Ensemble	4.969	3.764	0.804	18.819	7.473		59.248	40.298	0.881	14.051	4.458
Meta-Ensemble	4.642	3.370	0.826	18.509	6.978		64.441	45.384	0.860	14.809	5.015
Proposed FHE	**4.512**	**3.191**	**0.838**	**16.841**	**6.714**		**55.568**	**36.535**	**0.902**	**12.196**	**4.052**

↑ Greater is better. ↓ Lower is better.

**Table 5:** Impact of varying test set ratios on model performance across all forecasting models.

	Plant 1 (Capacity: 998 kW)							Plant 2 (Capacity: 369.85 kW)					
	**12% Testing Set**		**16% Testing Set**		**20% Testing Set**			**12% Testing Set**		**16% Testing Set**		**20% Testing Set**	
**Model**	**MAPE (%, ↓)**	**NMAE (%, ↓)**	**MAPE (%, ↓)**	**NMAE (%, ↓)**	**MAPE (%, ↓)**	**NMAE (%, ↓)**		**MAPE (%)**	**NMAE (%)**	**MAPE (%)**	**NMAE (%)**	**MAPE (%)**	**NMAE (%)**
RF	14.480	4.630	17.425	5.815	17.065	4.944		13.721	4.912	15.661	5.336	15.279	5.214
SVR	17.328	5.200	18.937	5.624	19.786	5.748		16.854	6.094	17.895	6.195	18.353	6.356
LGBM	14.773	4.613	16.972	4.929	17.577	5.014		14.284	4.966	14.940	5.031	15.719	5.239
XGB	16.417	5.308	18.142	5.354	18.618	5.438		14.668	5.265	15.512	5.324	16.053	5.464
Transformer	18.934	6.087	20.326	6.196	21.158	6.339		17.381	6.000	14.144	6.159	18.849	6.345
MLP	15.206	4.835	17.564	5.276	18.206	5.386		16.534	6.051	16.326	5.641	16.855	5.805
Bagging Ensemble	16.969	4.935	15.955	4.705	16.667	4.829		14.837	5.227	15.128	5.226	15.269	5.170
Meta-Ensemble	15.213	5.034	16.407	4.919	17.040	5.044		14.731	5.406	15.675	5.309	16.389	5.578
Proposed FHE	**12.923**	**4.356**	**14.600**	**4.459**	**15.079**	**4.585**		**12.332**	**4.686**	**13.486**	**4.843**	**14.079**	**4.968**
	Plant 3 (Capacity: 48.3 kW)							Plant 4 (Capacity: 905 kW)					
RF	18.508	7.001	19.486	7.701	19.443	7.326		14.116	4.558	14.223	4.634	14.536	4.722
SVR	19.875	7.498	20.574	7.918	20.471	7.561		17.645	5.344	17.380	5.333	17.660	5.403
LGBM	18.751	7.035	19.585	7.694	19.199	7.247		14.447	4.596	14.371	4.638	14.499	4.670
XGB	18.677	6.988	19.861	7.705	19.954	7.426		15.346	4.950	15.248	4.986	15.370	5.002
Transformer	22.701	8.434	22.953	8.734	22.842	8.418		16.397	5.138	16.553	5.232	16.639	5.256
MLP	21.674	8.542	22.069	8.974	21.542	8.419		16.537	5.212	16.370	5.208	16.610	5.268
Bagging Ensemble	18.819	7.473	18.779	7.406	18.909	7.256		14.051	4.458	14.027	5.641	14.255	4.548
Meta-Ensemble	18.509	6.978	19.679	7.768	19.672	7.375		14.809	5.015	14.686	5.055	14.978	5.038
Proposed FHE	**16.841**	**6.714**	**17.773**	**7.306**	**17.586**	**6.967**		**12.196**	**4.052**	**12.451**	**4.179**	**12.771**	**4.278**

↓ Lower is better.

**Table 6:** Model performance evaluated using time series cross-validation with shuffled temporal segments.

	Plant 1 (Capacity: 998 kW)				Plant 2 (Capacity: 369.85 kW)				Plant 3 (Capacity: 48.3 kW)				Plant 4 (Capacity: 905 kW)		
**Model**	**MAPE (%, ↓)**	**NMAE (%, ↓)**	$R^{2}$ (↑)		**MAPE (%)**	**NMAE (%)**	$R^{2}$		**MAPE (%)**	**NMAE (%)**	$R^{2}$		**MAPE (%)**	**NMAE (%)**	$R^{2}$
CV Iteration 1: 1 January 2019 to 13 September 2019															
RF	22.522	6.070	0.762		20.442	6.107	0.802		23.768	6.910	0.735		21.449	5.796	0.815
SVR	28.626	7.802	0.649		31.369	8.996	0.657		36.921	10.012	0.605		27.680	7.736	0.679
LGBM	21.946	5.853	0.761		22.186	6.225	0.798		26.108	7.357	0.720		17.706	5.156	0.843
XGB	24.903	6.620	0.726		22.711	6.628	0.762		31.497	8.139	0.677		23.354	6.402	0.774
Transformer	28.156	6.926	0.712		28.028	8.697	0.675		32.454	10.356	0.657		26.227	6.853	0.743
MLP	36.166	9.225	0.532		37.291	10.371	0.539		35.113	10.525	0.610		25.843	8.007	0.651
Bagging Ensemble	21.657	5.650	0.792		22.551	6.746	0.779		23.796	6.944	0.769		19.878	5.599	0.830
Meta-Ensemble	21.418	5.978	0.760		22.455	6.472	0.779		24.547	7.277	0.734		19.704	6.250	0.761
Proposed FHE	**19.138**	**5.236**	**0.816**		**19.968**	**5.801**	**0.816**		**21.911**	**6.425**	**0.779**		**16.565**	**4.795**	**0.869**
CV Iteration 2: 1 January 2019 to 25 May 2020															
RF	17.204	5.240	0.835		18.723	6.211	0.841		18.685	6.145	0.856		15.584	4.851	0.896
SVR	20.179	6.454	0.800		22.142	7.339	0.817		22.102	7.410	0.822		20.245	6.629	0.824
LGBM	17.183	5.176	0.848		18.307	6.202	0.848		18.709	6.280	0.857		14.444	4.559	0.908
XGB	17.917	5.478	0.827		20.053	6.593	0.829		19.968	6.586	0.848		15.659	4.878	0.889
Transformer	18.611	5.516	0.805		21.171	6.785	0.816		22.384	7.497	0.789		14.735	4.830	0.880
MLP	20.756	6.761	0.784		20.875	7.045	0.820		21.902	7.721	0.811		19.466	6.265	0.841
Bagging Ensemble	16.517	5.193	0.848		18.493	6.156	0.852		18.273	6.221	0.864		14.869	4.836	0.899
Meta-Ensemble	16.786	5.119	0.843		18.444	6.295	0.835		19.146	6.557	0.840		14.344	4.531	0.907
Proposed FHE	**15.059**	**4.518**	**0.874**		**16.706**	**5.453**	**0.868**		**16.019**	**5.415**	**0.885**		**13.454**	**4.265**	**0.915**
CV Iteration 3: 1 January 2019 to 5 Feberuary 2021															
RF	18.610	5.360	0.797		21.179	6.230	0.823		21.986	6.756	0.796		18.439	5.591	0.734
SVR	22.465	6.250	0.766		23.864	7.387	0.780		24.043	7.135	0.783		21.889	6.504	0.787
LGBM	19.515	5.491	0.796		21.841	6.437	0.814		21.865	6.789	0.792		18.879	5.732	0.772
XGB	20.108	5.666	0.770		22.249	6.522	0.809		22.177	6.850	0.787		20.084	6.201	0.729
Transformer	22.091	6.196	0.725		23.291	6.894	0.758		24.676	8.016	0.705		21.308	6.704	0.676
MLP	20.727	6.217	0.769		23.614	7.710	0.769		23.009	7.397	0.763		19.402	6.062	0.783
Bagging Ensemble	18.320	5.275	0.810		20.601	6.241	0.825		20.856	6.592	0.802		17.944	5.570	0.797
Meta-Ensemble	19.212	5.527	0.798		20.030	6.174	0.831		20.897	6.729	0.789		16.740	4.928	0.864
Proposed FHE	**16.649**	**4.691**	**0.839**		**18.773**	**5.575**	**0.848**		**19.743**	**6.044**	**0.827**		**16.684**	**4.967**	**0.846**
CV Iteration 4: 1 January 2019 to 18 October 2021															
RF	18.964	5.200	0.783		18.798	6.126	0.828		19.630	6.899	0.776		14.920	4.657	0.874
SVR	21.855	6.094	0.754		21.721	7.382	0.781		21.331	7.365	0.771		17.578	5.427	0.853
LGBM	19.219	5.229	0.779		18.960	6.122	0.835		20.187	7.032	0.769		14.748	4.562	0.880
XGB	20.945	5.712	0.745		20.097	6.505	0.813		21.325	7.515	0.742		15.635	4.871	0.864
Transformer	24.036	6.606	0.749		22.583	7.249	0.760		22.276	7.710	0.728		17.215	5.387	0.831
MLP	20.208	5.570	0.763		20.738	7.097	0.801		21.668	7.976	0.738		16.484	5.505	0.847
Bagging Ensemble	19.080	5.183	0.787		18.698	6.137	0.831		19.320	6.895	0.785		14.186	4.467	0.886
Meta-Ensemble	19.376	5.550	0.776		18.806	6.054	0.837		20.0645	7.056	0.776		15.222	5.020	0.853
Proposed FHE	**17.728**	**4.832**	**0.807**		**17.655**	**5.726**	**0.846**		**18.226**	**6.377**	**0.810**		**13.985**	**4.307**	**0.887**

↑ Greater is better. ↓ Lower is better.

## Data Availability

The meteorological data used in this study were obtained from the Korea Meteorological Administration (KMA) and are available at http://www.kma.go.kr/ (accessed on 15 July 2025). The PV power generation datasets used in the experiments are not publicly available due to commercial restrictions, but may be made available by the authors upon reasonable request.
